# Conditioned medium of IGF1-induced synovial membrane mesenchymal stem cells increases chondrogenic and chondroprotective markers in chondrocyte inflammation

**DOI:** 10.1042/BSR20202038

**Published:** 2021-07-02

**Authors:** Marlina Marlina, Rizki Rahmadian, Armenia Armenia, Jenifer Kiem Aviani, Ika Adhani Sholihah, Hanna Sari Widya Kusuma, Alya Mardhotillah Azizah, Nur Elida, Wahyu Widowati

**Affiliations:** 1 Faculty of Pharmacy, Andalas University, Jl. Limau Manis, Padang 25166, West Sumatera, Indonesia; 2 Faculty of Medicine, Andalas University, Jl. Limau Manis, Padang 25166, West Sumatera, Indonesia; 3 Biomolecular and Biomedical Research Center, Aretha Medika Utama, Bandung, Jl. Babakan Jeruk 2 no. 9, Bandung 40163, West Java, Indonesia; 4 Faculty of Medicine, Maranatha Christian University, Jl. Surya Sumantri no. 65, Bandung 40164, West Java, Indonesia

**Keywords:** Chondrogenesis, Col2, IGF1, Mesenchymal Stem Cells, MMP13

## Abstract

Recently, mesenchymal stem cells (MSCs) have been the most explored cells for cell therapy for osteoarthritis (OA) that can be obtained from various sources. Synovial membrane MSCs (SMMSCs) provide best potential for OA therapy, however they are not widely explored. Conditioned medium of SMMSCs (SMMSCs-CM) rich in growth factors and cytokines can inhibit apoptosis and increase chondrocytes cell proliferation. The aim of the present study was to determine growth factors content in SMMSCs-CM as well as the chondrogenic and chondroprotective markers expression in OA model after insulin-like growth factor (IGF)1-induced and non-induced SMMSCs-CM treatments. Chondrocyte cell line (CHON002) was induced by IL1β as OA model (CHON002 with IL1β (IL1β-CHON002)) and treated with SMMSCs-CM with or without IGF1 induction to determine its effectiveness in repairing OA cells model. ELISA was used to assay BMP2, fibroblast growth factor 18 (FGF18) and transforming growth factor (TGF) β1 (TGFβ1) levels in SMMSCs-CM, matrix metalloproteinase (MMP) 13 (MMP13) and a disintegrin and metalloproteinase with thrombospondin motif 4 (ADAMTS4) levels in OA cells model treated with SMMSCs-CM. RT-qPCR analyses were used to investigate the gene expression of SOX9, COL2, and COL10. CM from SMMSCs cultured and induced by IGF1 150 ng/mL was the most effective concentration for increasing the content of growth factor markers of SMMSCs-CM, which had successfully increased negative cartilage hypertrophy markers (SOX9 and COL2) and reduced hypertrophy markers (COL10, MMP13, and ADAMTS4). Preconditioning with IGF1 has better and very significant results in lowering MMP13 and ADAMTS4 levels. The present study supports IGF1 pre-conditioned SMMSCs-CM to develop a new therapeutic approach in OA improvement through its chondrogenic and chondroprotective roles.

## Introduction

Osteoarthritis (OA) is a joint degenerative disease affecting all joint tissues. Chondrocytes and extracellular matrix (ECM) compose the joint tissues. The survival of chondrocytes and homeostasis of cartilage ECM is maintained through cross-talk of several transcriptional factors including SRY-box 9 (SOX9), Runt-related transcription factor 2 (RUNX2), hypoxia-inducible factor (HIF1), and forkhead box O3 (FOXO3A) [1–3]. The balance of ECM composition in the cartilage is controlled by chondrocytes through both production of matrix molecules and their degradation by synthesizing proinflammatory cytokines, including interleukin (IL)-1 and tissue-destructive enzymes, such as matrix metalloproteinases (MMPs) and a disintegrin and metalloproteinase with thrombospondin motifs (ADAMTS) [4]. MMPs and ADAMTS work through degrading specific cartilage ECM components [5,6]. Extensive matrix degradation with higher rate than matrix synthesis causes irregularities in matrix composition with higher collagen type I (COL1) and COL10 to COL2 ratio, induction of cell apoptosis, and chondrocytes behavior alteration which marks the progression of OA [7].

Some studies stated that synovial membrane mesenchymal stem cells (SMMSCs) are one of the best candidates for cartilage damage repair [8–10]. SMMSCs’ ability to form cartilage can be predicted as they reside in the surrounding environment of cartilage, which makes them rich in COL2 and glucosaminoglycans (GAGs), the two key chondrogenic markers [11]. Moreover, these cells have no ability to develop into tumors, so that they are suitable for use in clinical practice [12].

Conditioned medium (CM) is a medium that contains stem cell derivative secretomes containing cytokines and growth factors that can repair tissue in various conditions [9,13,14]. CM may present an alternative to stem cell transplantation which has more advantages in lower risk of graft versus host disease, is easier in manufacturing, storage and handling, and also has longer shelf-life as well as higher availability as a ready-to-use biological product [15]. Bone morphogenetic protein 2 (BMP2) is the cytokine which initiates induction of chondrogenesis [16]. Fibroblast growth factor 18 (FGF18) can promote the development of cartilage, delay the degeneration of articular cartilage, and stimulate the potency for the regeneration of hyaline articular cartilage [17]. Transforming growth factors (TGFs) of β1 and β3, BMP2, BMP6 and BMP9, and insulin-like growth factors (IGFs) have been reported to induce or maintain chondrogenesis [18,19].

The use of growth factors, such as IGF1 provides enhancement in differentiation capacity of cells. IGF1 reduces the loss of chondrocytes and matrix integrity [20]. However, IGF1 injection does not give promising results for cartilage repair process as stated by some studies in preclinical and clinical trials through intra-articular injection. They found out that IGF1’s short half-life was the major cause for little to no therapeutic effects [21,22]. IGF1 preconditioning of MSCs may provide an alternative to enhance therapeutic potential in the MSC’s secretome and better strategy for cartilage repair process [23–25]. Therefore, this study aimed to determine the growth factors content and effect of CM from IGF1-induced SMMSCs towards chondrogenic and chondroprotective markers expression in OA cells model (CHON002 with IL1β (IL1β-CHON002)).

## Materials and methods

### SMMSCs collection and isolation

SMMSCs were collected from fresh knee joint biopsies of a patient with severe OA (Kellgren-Lawrence grade IV) in Dr. M. Djamil Hospital, Padang, Indonesia. SMMSCs treatment for severe OA and the subjects provided informed consent. The following experiments were performed after an approval was given by Research Ethics Committee, Faculty of Medicine, Andalas University, Padang, Indonesia (No. 226/KEP/FK/2019) [26].

### SMMSCs characterization

The isolated SMMSCs showed fibroblast-like morphology and plastic adherence as morphological property of MSCs. The cells also showed high level of CD90, CD44, CD73, CD105, while they showed low expression of hematopoietic lineage markers of CD11b, CD19, CD34, CD45, and HLADR. The MSCs also showed differential capacity into osteogenic, chondrogenic and adipogenic lineage, which marked their MSCs origins and multipotentiality [26].

### SMMSCs with IGF1 pre-conditioning

The fourth passage of SMMSCs was used for the experiment regarding their population doubling time (PDT) and cumulative population doubling (CPD) as presented in the previous experiment [26]. A total of of 5 × 10^5^ cells in 25-cm^3^ culture flask were cultured in minimum essential medium (MEMα) (Biowest, L0475-500) supplemented with 10% fetal bovine serum (FBS) (Biowest, S1810-500) with 1% (v/v) antibiotic–antimycotic (Biowest, L0010-100) and 1% (v/v) nanomycopulitine (Biowest, LX16-010) addition at 37°C and 5% CO_2_ for 2 d until the cells reached 80% confluence based on previous protocols [24–26].

The medium was replaced and treated with recombinant IGF1 (Biolegend, 590904) with concentrations of 0 ng/mL (negative control), 75, 150, and 300 ng/mL for 7 d and replacement every 2 d [24,27]. The CM of SMMSCs (SMMSCs-CM) was collected and centrifuged at 500×*g*, 4 mins and room temperature, the supernatant was filtered by 0.22-mm filter (TPP, 99722) stored at −80°C [24,25]. The BMP2, FGF18, and TGFβ1 secretions in SMMSCs-CM were measured using ELISA kit BMP2 (Elabsci, E-EL-H0011), FGF18 (Elabsci, E-EL-H5434), and TGFβ1 (Elabsci, E-EL-H0110) at 450 nm absorbance (Multiskan GO Thermo Scientific 51119300) [24,25]. IGF1 concentration that gave the highest BMP2, FGF18, and TGFβ1 level was used for further experiment.

### Induction of IL1β-CHON002 as *in-vitro* OA model

Human long bone, cartilage (CHON002 ATCC® CRL-2847™), was provided by Aretha Medika Utama, Biomolecular and Biomedical Research Center, Bandung, Indonesia as OA *in vitro* model. As many as 1 × 10^6^ cells of CHON002 were subcultured into T25 flask (SPL, 70075) incubated at 5% CO_2_ and 37°C in Dulbecco’s Modified Eagle’s Medium (DMEM) (Biowest, L0102-500) with 10% FBS, 1% (v/v) antibiotic–antimycotic, and 1% (v/v) nanomycopulitine. After the cells reached 80% confluence, the medium was replaced and treated with recombinant human IL1β (Biolegend, 579404) at 10 ng/mL concentration. The induction was carried out for 5 d as OA model according to previous protocol [24,25,27].

### IL1β-CHON002 treated with CM of IGF1-SMMSCs

Based on the IGF1 treatment on SMMSCs showed that 150 ng/mL IGF1 resulted in the highest level of BMP2, FGF18, and TGFβ1, furthermore for treating OA cells model using CM of 150 ng/mL IGF1-induced SMMSCs. The experimental groups were designed as follows: (1) CHON002 in MEMα complete medium (negative control); (2) CHON002 with IL1β induction (OA model); treatment groups: (3) OA model with administration of SMMSCs-CM 15% (v/v); (4) OA model with administration of SMMSCs-CM 30% (v/v); (5) OA model with administration of IGF1-SMMSCs-CM 15% (v/v); (6) OA model with administration of IGF1-SMMSCs-CM 30% (v/v). All cultures were incubated in 5% CO_2_ and 37°C environment for 7 d, the media were replaced every 2 d. SMMSCs-CM from the seventh day were taken for further analysis namely measuring the level of MMP13 and ADAMTS4 [24,25,27].

### Gene expression by real-time qPCR

CHON002 cells from all experimental groups were homogenized and their RNAs were extracted using Aurum Total RNA Mini kit (Bio-Rad, 7326820) in accordance with manufacturer’s protocols. cDNA synthesis was conducted using iScript cDNA synthesis kit (Bio-Rad, 1708890). The reaction was later incubated in thermal cycler with the following protocols: priming at 25°C for 5 min, reverse transcription at 46°C for 20 min, RT inactivation at 95°C for 1 min, and holding at 4°C. The cDNA was stored at −80°C until next assay was performed. SOX9, COL2, COL10 gene expressions were quantified through real-time qPCR for three replications per samples. The qPCR was done using SsoFast Eva Green Supermix kit (Bio-Rad, 1725200). Primer sequence as well as its product size and annealing temperature are presented in Table 1. Quantitative gene expressions were measured using Thermo Scientific PikoReal Real-time PCR System (Thermo Fisher). The gene expressions were quantified using ΔΔ*C*_t_ method relative to CHON002 expression [27,28]. Statistical analyses were carried on the Δ*C*_t_ values.

**Table 1:** Primers and RT-qPCR protocol

Gene symbols	Primer sequence (5′–3′) Upper strand: sense Lower strand: antisense	Product size (bp)	Annealing (°C)	References
*GAPDH*	GAGCCACATCGCTCAGACAC	150	51	NM_001289745.3
	CATGTAGTTGAGGTCAATGAACG			
*SOX9*	CACACAGCTCACTCGACCTTG	76	51	Widowati et al. [24]
	TTCGGTTATTTTTAGGATCATCTCG			
*COL2*	TTTCCCAGGTCAAGATGGTC	377	53	Widowati et al. [24]
	CTGCAGCACCTGTCTCACCA			
*COL10*	CCCTTTTTGCTGCTAGTATCC	468	51	NM_000493.4
	CTGTTGTCCAGGTTTTCCTGGCAC			

### Quantification of MMP13 and ADAMTS4 levels

Secretions of MMP13 and ADAMTS4 in OA model treated with SMMSCs-CM were assessed using ELISA Kit for MMP13 (Elabsci, E-EL-H0134) and ADAMTS4 (Elabsci,E-EL-H0266). The procedure was in accordance with manufacturer’s protocols. The sample absorbance at 450 nm was measured with three replications using microplate reader [24,25].

### Statistical analysis

The significance differences between various IGF1 concentration inductions were determined from ANOVA with post-hoc Tukey’s HSD analysis. The significance between OA treatment groups were determined from Independent *t* test or Mann–Whitney U test based on data normality. All statistical analyses were carried out on 95% confidence interval.

## Results

### BMP2, FGF18, and TGFβ1 levels in SMMSCs-CM

**Figure 1:**
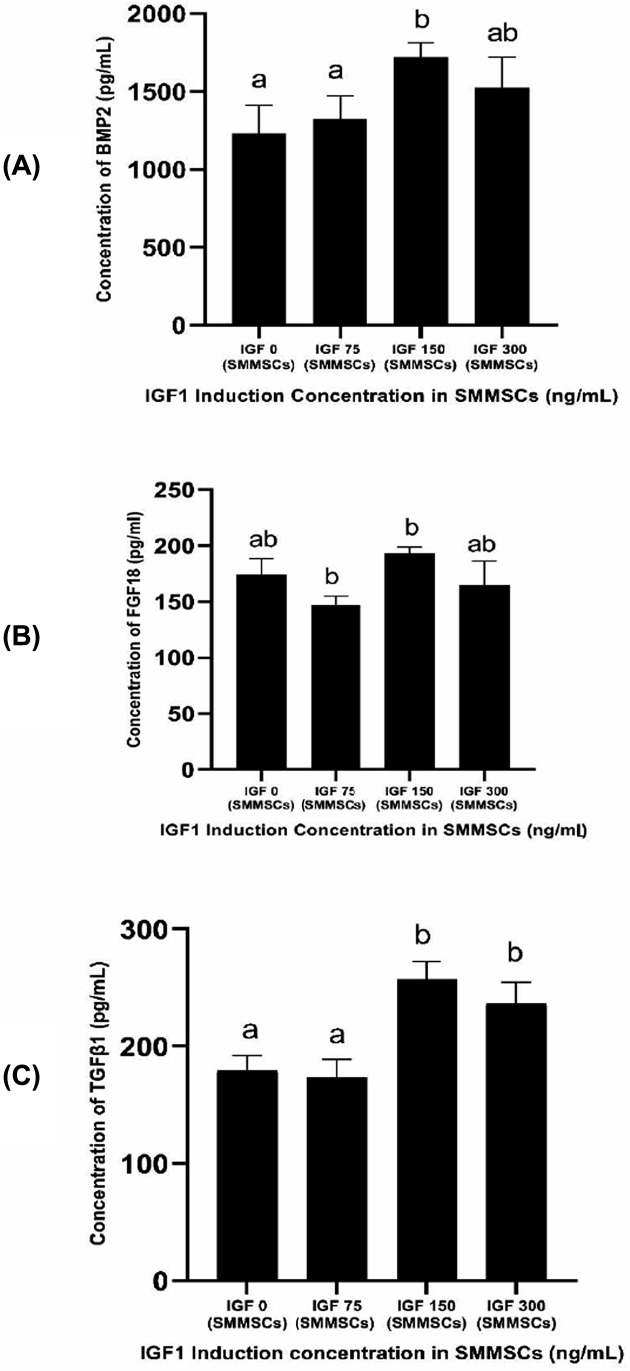
Effects of various concentrations of IGF1 towards BMP2, FGF18, and TGFβ1 levels of SMMSCs-CM (**A**) Concentration of BMP2 (pg/mL) in IGF1-induced SMMSCs-CM. (**B**) Concentration of FGF18 (pg/mL) in IGF1-induced SMMSCs-CM. (**C**) Concentration of TGFβ1 (pg/mL) in IGF1-induced SMMSCs-CM. *The data were presented as a histogram of mean ± standard deviation. Different letters (a, ab, b) marked significant difference among treatments towards BMP2 level (A). Different letters (a, ab, b) marked significant difference among treatments towards FGF18 level (B). Different letters (a, b) marked significant difference among treatments towards TGFβ1 level (C). The data were analyzed using ANOVA followed by Tukey’s HSD post hoc test (*P*<0.05).

IGF1 treatment in various concentrations showed that 150 ng/mL IGF1 induced the highest level secretion of BMP2, FGF18, and TGFβ1 in SMMSCs, which was significant compared with other treatments (*P*<0.05) (Figure 1A–C). Thus, CM of IGF1-SMMSCs with this concentration was the one that would be used for further treatment in OA model.

### SOX9, COL2, and COL10 gene expression levels in OA model

**Figure 2:**
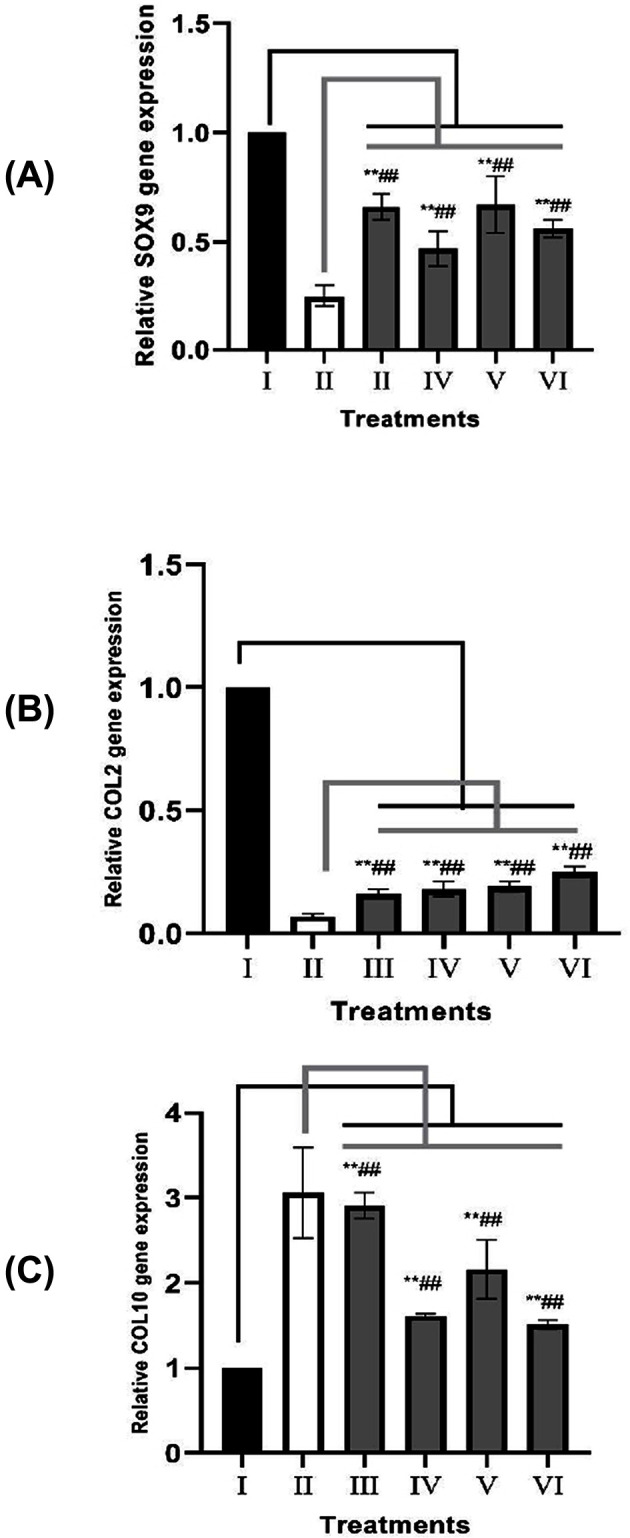
Effects of IGF1-induced SMMSCs-CM toward SOX9, COL2, and COL10 gene expressions on OA model (**A**) Relative SOX9 gene expression in IGF1-induced SMMSCs-CM. (**B**) Relative COL2 gene expression in IGF1-induced SMMSCs-CM. (**C**) Relative COL10 gene expression in IGF1-induced SMMSCs-CM. (I) CHON002 (healthy cells as negative control), (II) IL1β-CHON002 (OA model), (III) OA model+SMMSCs-CM 15% (IV), OA model+SMMSCs-CM 30% (V) OA model+IGF1-SMMSCs-CM 15% (VI) OA model+IGF1-SMMSC-CM 30%. *The figure above shows gene expressions relative to CHON002 (negative control). Data are presented as mean ± standard deviation. Double star sign (**) marks statistical difference compared with negative control group at 0.01 significance level, double hashtag (##) marks statistical difference compared with OA model at 0.01 significance level.

IL1β induction resulted in significant reduction in SOX9 compared with negative control (*P*=0.001). All treatments successfully significantly increased SOX9 gene expression compared with OA model (*P*<0.012) (Figure 2A). The IGF1 pretreated on SMMSCs shows no differences compared with those of non-induced groups (*P*=0.589). Low concentration of SMMSCs-CM (15% v/v) significantly causes higher SOX9 expression (*P*=0.014).

IL1β induction resulted in significant reduction in COL2 expression compared with negative control (*P*=0.000). All treatments have successfully significantly increased COL2 gene expression in OA model (*P*=0.010) (Figure 2B). IGF1 preconditioning causes significant effect compared with those of non-induced groups (*P*=0.041). High concentration of SMMSCs-CM (30% v/v) causes no difference in COL2 expression (*P*=0.094).

CHON002 induced with IL1β shows significant COL10 overexpression compared with negative control (*P*=0.000). SMMSCs-CM without IGF1 preconditioning at low concentration (15% v/v) cannot reduce COL10 overexpression (*P*=0.740), however high concentration of SMMSCs-CM significantly reduces COL10 expression (*P*=0.003, *P*=0.004) (Figure 2C). The higher concentrations of SMMSCs-CM has significantly given better results in both IGF1 induction and non-induced groups (*P*=0.002). IGF1 induction on SMMSCs causes no difference compared with that of non-induced group (*P*=0.180).

### MMP13 and ADAMTS4 levels in OA model

**Figure 3:**
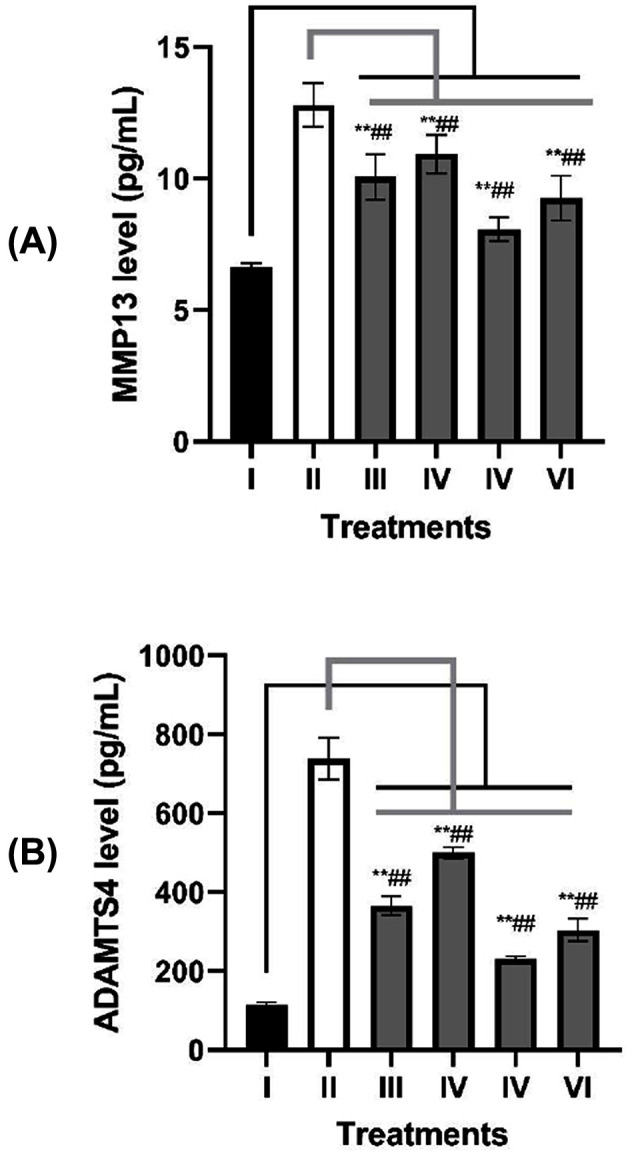
Effects of IGF1-induced SMMSCs-CM toward MMP13 and ADAMTS4 levels on OA cells model (**A**) MMP13 level (pg/mL) on OA cells model. (**B**) ADAMTS4 level (pg/mL) on OA cells model. (I) CHON002 (healthy cells as negative control), (II) IL1β-CHON002 (OA model), (III) OA model+SMMSCs-CM 15% (IV), OA model+SMMSCs-CM 30% (V) OA model+IGF1-SMMSCs-CM 15% (VI) OA model+IGF1-SMMSC-CM 30%. *Data are presented as mean ± standard deviation. Double star sign (**) marks statistical difference compared with negative control group at 0.01 significance level, double hashtag (##) marks statistical difference compared with OA model at 0.01 significance level.

IL1β induction has resulted MMP13 level significant increase compared with negative control (*P*=0.000). All treatments have very significantly decreased MMP13 level on OA model (*P*<0.010) (Figure 3A). IGF1 preconditioning has successfully lowered MMP13 significantly compared with those of non-induced groups (*P*=0.005).

ADAMTS4 level significantly increased in IL1β OA model compared with negative control (*P*=0.000). All treatments have resulted in very significant reduction in ADAMTS4 level in OA model (*P*<0.005) (Figure 3B). IGF1 induction on SMMSCs gave a very strong effect in lowering ADAMTS4 compared with those of non-induced groups (*P*=0.002). There was no effect of the concentration difference between IGF1-SMMSCs-CM (*P*=0.084). The result shows that SMMSCs-CM can suppress the levels of MMP13 and ADAMTS4 which work as matrix degradation. IGF1 preconditioning gives better results to down-regulate MMP13 and ADAMTS4 levels.

## Discussion

Preconditioning of MSCs culture has been reviewed to upscale the production of specific factors in the MSCs secretome [23]. IGF1 stimulation has been reported to up-regulate several growth factors including TGFβ [29] and BMP [30]. Our study also shows that IGF1 preconditioning in SMMSCs has successfully increased secretion of TGFβ1, BMP2, and FGF18. CM contains various growth factors and tissue regenerative agents, which were secreted by the stem cells. The fact that stem cells secrete various growth factors was also shown by various proteomics studies, which revealed the presence of various growth factors and other cytokines in the CM [31]. While BMP2 was infused at high concentrations (1.5 mg/mL), low concentrations of supplemented growth factors were sufficient to synergize with the components within MSCs-CM and further promoted bone regeneration [32]. Data from transwell coculture systems indicated that MSCs promoted the expressions of chondrogenic genes in chondrocytes in the presence of FGF18. Ectopic formation confirmed that FGF18 treatment restored the responsiveness of OA chondrocyte to MSCs *in vivo* [33]. According to a research by Zhang et al. (2014), FGF18 can increase the responsiveness of OA chondrocytes to the trophic effects of MSCs [33].

SOX9 is an important transcription factor in chondrocytes because of its role in the differentiation of chondrocytes and the regulation of ECM deposition, especially COL2. SOX9 inhibition causes a decrease in the differentiation of new chondrocytes to replace chondrocytes that undergo apoptosis as a result of mechanical and inflammatory stress. The decrease in the rate of ECM deposition also exacerbates the imbalance of the anabolic and catabolic processes in the cartilage which further inhibits the regenerative power of the joint cartilage [34]. In some recent studies, it has been shown that OA cartilage has a low expression of SOX9 [35–38]. IL1β has been known to reduce SOX9 and COL2 gene expressions as stated by some studies [24,39–42]. The down-regulation mechanism is caused by nuclear factor-κB (NFκB) inflammatory pathway [39–41]. The cAMP response element-binding protein (CREB) and SP1 transcription factor, two proteins that bind to SOX9 transcription binding site that have positive action on SOX9 transcription, are blocked during inflammation induced by IL1. Another protein proposed to participate in SOX9 transcriptional activity is SMAD3, a mediator from TGFβ pathway [43].

COL2 is a chondrogenic gene marker associated with ECM in joint cartilage. In OA, the early changes in articular cartilage are characterized by the loss of proteoglycan and a reduction in the expression of COL2 gene without any changes to the regularity of the structure of the articular tissue [44]. COL2 is one of SOX9 target protein and in the initial chondrogenesis process, SOX9 expresses ECM proteins, such as collagen type 2 α 1 (COL2a1) [45]. IL1β has markedly reduced COL2 expression to almost none in our experiment. SOX9 protein negatively regulates *RUNX2* gene by binding to BAPX1 in proliferating chondrocytes. During inflammation, RUNX2 binding to SOX9 represses the COL2a1 expression [46]. COL2 expression is not only influenced by SOX9, but also mediated by NKX3-2 transcription factor [47]. BMP7 can up-regulate reduced-BAPX-1/NKX3-2 level in IL1β OA model [48]. Our result shows that SMMSCs-CM treatment can markedly increase COL2 expression. We hypothesize that this may be because of BMP as well as TGFβ action in the SMMSCs-CM, which can be shown by dose-dependent trend and better result in IGF1-induced treatment. However, compared withr healthy cell control, the COL2 level is still remarkably lower. It is epithelium-specific ETS (ESE)-1 transcription factor that keeps the persistent NFκB-dependent COL2a1 promoter inhibition by IL1β. This characteristic is found in patients with OA [49].

Type X collagen (COL10) is a hypertrophic chondrocyte marker that is not usually found in adult articular cartilages but will be detected at some stages in OA. COL10 expression will increase significantly in the final stage of OA compared with the initial stage of OA. MSCs-CM which is rich in growth factors will suppress hypertrophic cartilage markers which are characterized by the fall of COL10 expression [50]. We found that the expression of the COL10 gene increased after the addition of 1L1β. IL1β acts through P38 MAPK in RUNX2 protein phosphorylation [51]. IL1β-induced inflammation also reduces BAPX1 gene expression which is the inhibitor of RUNX transcription [48]. Our treatment using SMMSCs has successfully reduced COL10 expression in OA model. In proliferating chondrocytes, SOX9 together with other transcription factors bind to the COL10a1 promoter region that causes gene repression. However during inflammation, RUNX2 binding to SOX9 releases SOX9 inhibitory binding that will cause COL10a expression [46]. Our result shows negative correlation between COL10 and SOX9 expression (r = −0.362, *P*=0.039) which marks that SOX9 expression negatively regulates COL10. TGFβ through SMAD3 signaling can counteract RUNX2 by direct binding deactivating this transcription factor and by recruiting silencing histone class II deacetylase to the responsive genes, such as COL10 and MMP13. BMP2 signaling stabilizes SMAD3-TGFβ pathway [52]. Moreover, BMP7 signaling pathway through NKX3-2 has a negative effect toward RUNX2 level [48]. FGF, mainly FGF9 and FGF18, acts mainly through FGF receptor 3 (FGFR3) by inhibiting the initiation of hypertrophy through MAPK signaling [53].

Cleavage of COL2 is considered as a critical early event of OA. Initial cleavage of COL2 is performed by MMP13 [54]. Figure 3A shows that IL1β-CHON002 has high MMP13 levels. It has been known that IL1β mediates MMP13 and ADAMTS4 through NFκB pathway [55]. Moreover RUNX2 is needed for the transcription mediated by P38-MAPK pathway [51,56].

**Figure 4:**
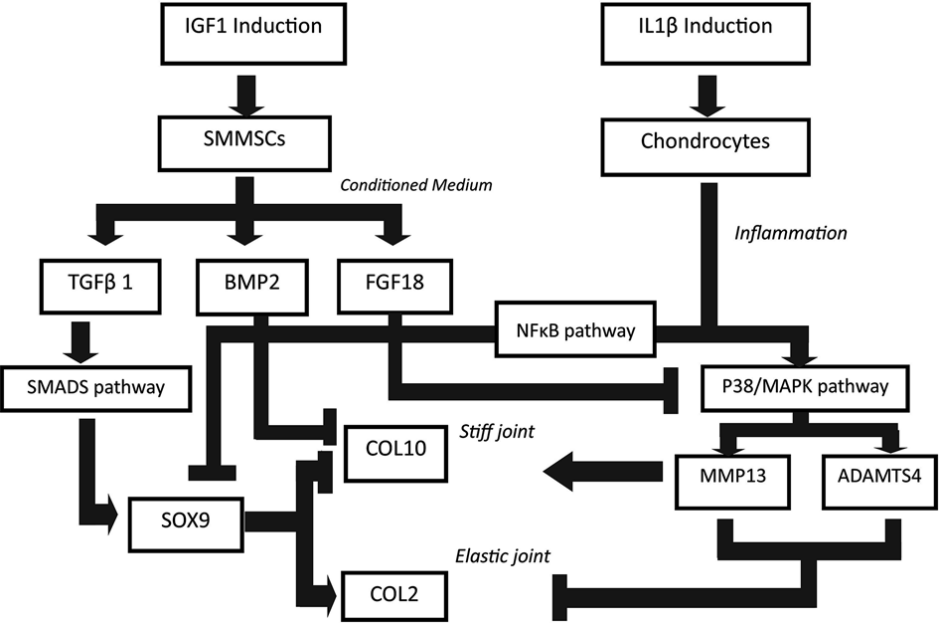
Proposed mechanism on cartilage repair and chondroprotective effects of IGF1-induced SMMSC-CM therapy SMMSCs secrete many growth factors, such as TGFβ1, FGF18, and BMP2 whose concentrations increase with the IGF1 induction. These factors are contained in the SMMSCs-CM. IL1β induces chondrocyte inflammation and triggers OA involving two different pathways: the NFκB and P38-MAPK. During inflammation, the NFκB protein plays a role in decreasing in SOX9 gene expression. SMMSCs treatment up-regulate SOX9 expression mainly through TGFβ1 action via SMAD2/SMAD3 pathway. SOX9 acts as an activator of COL2 gene, and an inhibitor of COL10. During IL1β-induced inflammation of chondrocytes in which MMP13 and ADAMTS4 are up-regulated through P38/MAPK pathway. SMMSCs treatments act in alleviating the OA symptoms through: (1) BMP2 down-regulates COL10; (2) FGFs, mainly through FGF18 that acts by inhibiting MMP13 and ADAMTS4 synthesis through P38/MAPK pathway. COL2 deposition results in healthy and elastic joint. Meanwhile, COL10 causes stiffness in the joint, which is one characteristic of OA.

Based on our results and literature research, we proposed the possible mechanism of OA as well as SMMSCs-CM and IGF1 chondroprotective role. The mechanism is shown in Figure 4. Overall, the growth factors contained in the SMMSCs-CM provides chondroprotective role by inhibiting matrix degradation enzymes and alleviates hypertrophic marker and chondrogenic role by increasing COL2 level.

## Conclusion

The concentration of 150 ng/mL of IGF1 for 7-day incubation is the optimal growth factor markers including BMP2, FGF18, and TGFβ1 on SMMSCs-CM. SMMSCs-CM has increased negative cartilage hypertrophy markers (SOX9 and COL2) and reduced hypertrophy markers (COL10, MMP13, and ADAMTS4) in OA model. SMMSCs preconditioning with IGF1 have better results in lowering the levels of MMP13, ADAMTS4, and gene expression of COL10, as well as increasing gene expressions of SOX9 and COL2. Overall, SMMSCs-CM has the potential as an alternate therapy for OA through its chondroprotective and chondrogenic role. Further research is needed to prove the potential of IGF1-induced SMMSCs on OA animal model.

## Data Availability

The datasets used and/or analyzed during the current study are available from the corresponding author on reasonable request.

## Abbreviations

ADAMTS: a disintegrin and metalloproteinase with thrombospondin motif
BMP2: bone morphogenetic protein
CM: conditioned medium
COL2a1: collagen type 2 α 1
COL10: type X collagen
ECM: extracellular matrix
ETS: E26 Transformation-specific
FBS: fetal bovine serum
FGF18: fibroblast growth factor 18
IGF: insulin-like growth factor
IL: interleukin
MEMα: minimum essential medium
MMP: matrix metalloproteinase
MSC: mesenchymal stem cell
NFκB: nuclear factor-κB
OA: osteoarthritis
RT-qPCR: Real Time Quantitative PCR
RUNX2: Runt-related transcription factor 2
SMMSC: synovial membrane MSC
SMMSCs-CM: conditioned medium of SMMSCs
